# Supplementary material for: Patterns of Intron Gain and Loss in Fungi
Source: PLoS Biol. 2004 Nov 30;2(12):e422. doi: 10.1371/journal.pbio.0020422 (PMC532390; doi:10.1371/journal.pbio.0020422)
Supplement: Table S1 — Also available at http://genes.mit.edu/NielsenEtAl/. (4.3 MB ZIP). [file pbio.0020422.st001.zip › NielsenEtAl/html/562.html]

AN1537.1.NCU03048.1.MG04760.1.FG00512.1


```
 CLUSTAL W (1.82) Multiple Sequence Alignments - Introns Inserted


Sequence 1: NCU03048.1	432 aa
Sequence 2: FG00512.1	435 aa
Sequence 3: MG04760.1	486 aa
Sequence 4: AN1537.1	448 aa
Alignment Length: 514 aa
Number Identitical Residues: 89 aa
Alignment Score (without introns) 6087


MG04760.1 	-MSSAEDQDLLARIGQLAG1QINRHKSQQAGVGDSAPAPNPTPSHHYHPYHCMFSRHHYT
NCU03048.1	MTMSAEEHELMERIARVAG~QINQHKNQQAGL--VPPQSSHAPPHHRTSVEYHNSNRAGI
FG00512.1 	--MSEEERELLARIGQLAG1QINRHKNGQTGP--RAPPNLH-PAHHRRMLGFELQRRSTA
AN1537.1  	---MSEDQDLMARISQLAG1QINRFKNGNTPV-----QSAHNEMHSNSHVSRHTSYRG--
          	     *:::*: **.::** ***:.*. ::              *         . :   

MG04760.1 	GHRFDKLQAAGHASQPWGRGRGSYRSRGARGGHVAP-HRHRTLILNTNTTSDVTGDGTKS
NCU03048.1	DGLEDSGYSG-----SWRHQRGGYPYPT-RAHYTKPAHRHRTLVLNG---------ASQQ
FG00512.1 	NAHLDN---------SYRHASSPYPARHNRIGRPPAAHQHKTLHLN----------ADSS
AN1537.1  	-----------------RPGWAPYRGRPYGRGRGAAPHRHRTLILNN---SATPASKSST
          	                     . *           ..*:*:** **    :  ...  . 

MG04760.1 	GAPQSAGLASASSPFSTSPSWVTKKDRNLQLINSAVYERESLARSIGAAEARRQKFKMLN
NCU03048.1	GKAGPP--SSGAALDSSPSSWVAKNDRHLQIINSSIYHKEAQARTRAIEQTLRQKQQQRD
FG00512.1 	NASRSA--SSGAET---PPGWVSRNDRHRQLINANVYERDTHNRAKAIEQTHQNKINGYR
AN1537.1  	PPDGMAIDTDENSRSATPNAWVTKHARHMQLINSAVYDKEAQKRVKALEETRKAKAQKKA
          	     .  :.     ::. .**::: *: *:**: :*.:::  *  .  :: : * :   

MG04760.1 	QRERAKLLRHFQSGSHATMARPATNA-----SATQYEVIIEGIKFHVAKNGSKLVKVAD1
NCU03048.1	DLERTKLINHLHRAGDRSAAAPN-----------QYELTVQGIRFAVTKNGSKLVKIPG1
FG00512.1 	QREKTQFNEFLKHQAGASSAQTNP--------AGQNELTVQGVQFRVMDGGKKLVKIPD1
AN1537.1  	QIEQAKVLQFAHGAGRQFSSSSNPQVSAAGESHGEYQITLNDIPFRVSRGGGKLIRVSS~
          	: *:::. .. :  .    : . .. :::..:  : :: ::.: * *  .* **:::.. 

MG04760.1 	N-------------------NAPKATPKQAVVGGVKFLRSRNGNMVRHDI~VKAQR~YDP
NCU03048.1	DL-----------------NSGP--TPKMAIVGGVKFYRSKNGNLYRHGI~VKAQR2---
FG00512.1 	AP-----------------NSSSSRTPKTATVAGVRFYRTKTGNLVASRI~VNDHR2R--
AN1537.1  	ATPVLSALIERAGLQIVDDPNTANNTPKRVKVAGVTFVRSKNGNLHRLGA0SALGG~Q--
          	  .  ::  . :. .  .. . .. *** . *.** * *::.**:               

MG04760.1 	RPPSCGTLTEKFSRQNGPVRKVQTPCRMFSTTG1SCTKG~PLCRYQHDPLKVAICKDFLL
NCU03048.1	------------------FRNLNRHHVEMTGS-~----G1PRCRYIHDPAKVAICKDFLQ
FG00512.1 	---------------SGVVKKIDQLCKIFSTTG1SCTKG~PRCRYIHDPSKVALCKNILK
AN1537.1  	-------------------EKGRALPKIHYDG-~-----~--YQFFPQALRIPFVN----
          	                   .:                      ::  :. ::.: :    

MG04760.1 	KGDCIGGDSCDLSHEPTPER~TPACLHFAKGNCNNPQCRYTHVQVTPGSLVCRDFGIYGY
NCU03048.1	QEECINGDSCDLSHELSAER~TPTCLHFIKDSCTKPDCKFTHAKVSPAASVCREFGLYGY
FG00512.1 	DGQCVNGELCDLSHDMTPER~TPNCLHFAKGHCAKDDCPYTHSRAPPAALVCRSFGFNGY
AN1537.1  	---CSPSQVQRVMLTDISSR2SPACVHFLRGRCSNPECRYAHVRVTPGAPVCRAFATLGY
          	   *  .:   :     ..* :* *:** :. * : :* ::* :..*.: *** *.  **

MG04760.1 	CNKGSNCEDRHVFECPDFSNTGHCKIKGCKLTHRERASVLRKAVASKDEDDSEMEDVSSD
NCU03048.1	CEKGASCINRHVFECPDFSNTGVCNTKGCKLPHRERASVLRRVNAR---DDAEMEDLSSD
FG00512.1 	CEKGADCTERHVFECPDFSNTGRCKIKGCKLPHRERASVLRNRTNA---DGEPLGDVSSD
AN1537.1  	CDKGETCEERHVHECPDYANTGVCKKKHCRLPHVDRAGQIRKNAGPKEGEGDDESDASSE
          	*:**  * :***.****::*** *: * *:*.* :**. :*.    .. :.    * **:

MG04760.1 	ED-DESVDSNDVDSDAVDEFLGEDEKSDV-DPDEFKEYIQL
NCU03048.1	---DDSVDSDDIDSDDVDEFVGQDDDPDL-DFAEQKDFIKF
FG00512.1 	---DEAADSDDVDSDEVAEFIDADSDLS--DFEEQKDFLSI
AN1537.1  	EEEFDEIGSDDVDSDYLSDEGELIEGTETGEVSKQQDFIRL
          	... :  .*:*:*** : :     .  . .:  : :::: :
```
